# The impact of antibiotics on the gut microbiota of children recovering from watery diarrhoea

**DOI:** 10.1038/s44259-024-00030-x

**Published:** 2024-04-22

**Authors:** Son-Nam H. Le, Chau Nguyen Ngoc Minh, Paola Florez de Sessions, Song Jie, Chau Tran Thi Hong, Guy E. Thwaites, Stephen Baker, Duy Thanh Pham, Hao Chung The

**Affiliations:** 1 Oxford University Clinical Research Unit, Ho Chi Minh City, Vietnam; 2 School of Biotechnology, International University, Vietnam National University, Ho Chi Minh City, Vietnam; 3 Genome Institute of Singapore, Singapore, Singapore; 4 Centre for Tropical Medicine and Global Health, Nuffield Department of Clinical Medicine, University of Oxford, Oxford, United Kingdom; 5 Department of Medicine, Cambridge Institute of Therapeutic Immunology and Infectious Diseases (CITIID), University of Cambridge, Cambridge, United Kingdom; 6 Saw Swee Hock School of Public Health, National University of Singapore, Singapore, Singapore

**Keywords:** Microbiome, Antibiotics, Gastroenteritis

## Abstract

Infectious diarrhoeal diseases remain a substantial health burden in young children in low- and middle-income countries. The disease and its variable treatment options significantly alter the gut microbiome, which may affect clinical outcomes and overall gut health. Antibiotics are often prescribed, but their impact on the gut microbiome during recovery is unclear. Here, we used 16S rRNA sequencing to investigate changes in the gut microbiota in Vietnamese children with acute watery diarrhoea, and highlight the impact of antibiotic treatment on these changes. Our analyses identified that, regardless of treatment, recovery was characterised by reductions in *Streptococcus* and *Rothia* species and expansion of *Bacteroides*/*Phocaeicola*, Lachnospiraceae and Ruminococcacae taxa. Antibiotic treatment significantly delayed the temporal increases in alpha- and beta-diversity within patients, resulting in distinctive patterns of taxonomic change. These changes included a pronounced, transient overabundance of *Enterococcus* species and depletion of *Bifidobacterium pseudocatenulatum*. Our findings demonstrate that antibiotic treatment slows gut microbiota recovery in children following watery diarrhoea.

## Introduction

Infectious diarrhoeal diseases remain a leading cause of death in children under five in low- and middle-income countries (LMICs)^1,2^. It is defined as having more than three loose or watery stools within 24 h and is caused by bacteria, viruses, and/or parasites. Apart from direct assaults by the pathogen, the gut environment also undergoes increased bowel movements and fluid secretion during diarrhoea^3^. This exerts a significant perturbation to the inhabiting microbial communities (microbiota) and the encompassing environment (gut microbiome). Furthermore, children with diarrhoea are usually treated with oral rehydration, probiotics, and in some instances, antibiotics^4,5^. These treatments add another layer of complexity to the interaction with the gut microbiota by inducing indirect killing (antibiotic)^6^ or replenishing (probiotic) effects^3^.

The healthy gut microbiota in children is predominated with obligate anaerobes belonging to the three phyla: Actinobacteria, Bacteroidetes, and Firmicutes^7^. The exact taxonomical composition is dependent on factors as varied as the birth-delivery method, breastfeeding, age, time of weaning and nutritional status^8–10^. We previously showed that some of these factors could help shape the gut microbiota state during the early phase of diarrhoea (Day 1–5 of dysbiosis), which was predominated by the facultative anaerobes *Escherichia* and *Streptococcus*^3,11,12^. Previous studies employing shotgun metagenomic sequencing on Bangladeshi children with diarrhoea documented the gut microbiome succession during the course of disease^3,13^. While the transiently oxygenated gut facilitates the expansion of facultative anaerobes in the early phase, diarrhoea recovery is marked by the growth of anaerobic *Bacteroides* during the mid-phase (Day 7–10) and of a highly diverse community of Firmicutes at the late phase (Day 14 onward)^3^. Nevertheless, such changes are predicted to be highly variable between individuals, notably in response to the treatment during diarrhoea. Previous studies, though incorporating longitudinal design, have not addressed the impact of treatment on the gut microbiome dynamic during diarrhoea recovery.

Antibiotics are known to destabilise the gut microbiome in healthy individuals^8,14,15^. The gut microbiome acts as a major reservoir for antibiotic-resistant bacteria^16,17^. Therefore, antibiotic treatment does not only diminish species richness but also give rise to adapted resistant bacteria, frequently through the horizontal transfer of antimicrobial resistance (AMR) genes within and between species^18–20^. However, it is unclear to what extent antibiotic treatment affects the gut microbiome in children with diarrhoea, especially during recovery. To investigate this question, we studied children enroled on a previously published trial of probiotics for diarrhoea treatment^21^.

## Results

Our study utilised stool samples collected from a longitudinal cohort of a recent randomised control probiotic trial in Vietnam (Longitudinal study), demonstrating that supplementation with *Lactobacillus acidophilus* did not shorten diarrhoeal duration^21^. The collected samples (*n* = 218) here originated from 90 diarrhoeal patients, mostly belonging to the probiotic arm (*n* = 85/90) (Table 1). The study recruited patients with acute watery diarrhoea, who provided stool samples at hospital admission (day 1–D1), then at day 7 (D7) and 14 (D14). From D7, most patients ceased to have diarrhoea (*n* = 85/90). The majority of patients were diagnosed with Rotavirus or Norovirus infections (mono- or mixed, >80%) using real-time PCR. Nearly one-third of patients (*n* = 28/90) were breastfed, and the median age was 16 months (interquartile range [IQR]: 11.80–21.75). Nearly 40% of patients (*n* = 35/90) were treated with antibiotics, most commonly ciprofloxacin or 3^rd^ generation cephalosporins. Antibiotics were prescribed to patients during the first week (median Day 2; range: Day 1–6), and the median duration of therapy was 6 days (range: 3–10). The median duration of hospitalisation was four days (IQR: 2–5), and the median diarrhoea duration recorded in the hospital was 44 h (IQR: 21–74). Stool samples were subjected to DNA extraction and 16S rRNA gene amplification and sequencing. Additionally, we included 16S rRNA sequencing data from our previous cross-sectional study on the diarrhoeal microbiota of Vietnamese children, which employed a similar sequencing and analysis approach^12^. A preliminary quality check showed that there was no apparent study-wise clustering when combining these two datasets (Supplementary Fig. 1).

**Table 1:** Demographic and clinical data of the longitudinal study

	Total (% or IQR)
Sex	
Male	60 (66.67)
Female	30 (33.33)
Age months	
Median (IQR)	15.95 (11.80–21.75)
Infection types^a^	
Rotavirus only	33 (36.67)
Virus + Bacteria	21 (23.33)
Virus + Parasite	9 (10.00)
unknown	8 (8.89)
Norovirus	7 (7.78)
Bacteria only	5 (5.56)
Adenovirus	2 (2.22)
Norovirus + Rotavirus	2 (2.22)
Adenovirus + Norovirus + Rotavirus	1 (1.11)
Adenovirus + Rotavirus	1 (1.11)
Adenovirus + Norovirus	1 (1.11)
Breastfeeding	
No	62 (68.89)
Yes	28 (31.11)
Antibiotic treatment	
No	55 (61.11)
Yes	35 (38.89)
Probiotic treatment	
Yes	85 (94.44)
No	5 (5.56)
Hospitalisation duration (days)	
Median (IQR)	4.00 (2.00–5.00)
Diarrhoea duration (hours)	
Median (IQR)	43.50 (21–74)
wfa z-score	
Median (IQR)	−0.04 (−0.91–1.16)

^a^Pathogens: Virus: Adenovirus, Norovirus, Rotavirus; Bacteria: diarrheagenic E. coli, Campylobacter, Salmonella, Shigella; Parasite: Cryptosporodium, Giardia.

We found that antibiotic treatment was the sole significant predictor of hospitalisation duration (Multiple Linear Regression, *p*-value = 0.001). Antibiotic treatment was linked to prolonged hospitalisation (mean difference of 1.8 days, Wilcoxon test, *p* = 0.002) (Supplementary Fig. 2) but was not associated with different diarrhoeal etiologies (Chi’s square test, *p* = 0.81). Since antibiotics are known to impact the gut microbiome^22,23^, we set out to investigate their influences on the longitudinal recovery of the gut microbiota in our cohort. Due to the small sample size of antibiotic-treated group, we did not divide the study population by antibiotic classes for downstream analyses.

### Antibiotic treatment delayed increases in alpha-diversity of the gut microbiota

**Fig. 1:**
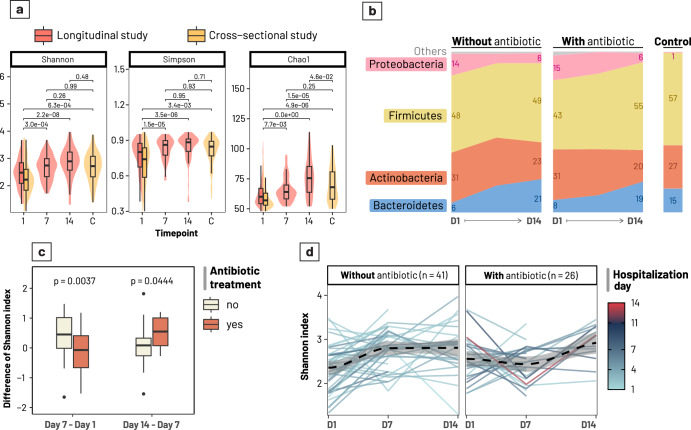
Temporal changes in alpha-diversity and phylum abundances in the gut microbiome of children recovering from diarrhoea. **a** Alpha diversity indices (Shannon, Simpson, and Chao1) calculated and grouped by timepoints (Days 1, 7, 14 since hospitalisation by diarrhoea) and coloured by studies (longitudinal – coral, cross-sectional – gold). Group C denotes non-diarrhoea controls (see Legend). Pairwise comparisons were performed using Analysis of variance (ANOVA) with Tukey post-hoc tests, with p-values attached. **b** Relative abundances of different phyla, calculated as the mean value separately for each timepoint and antibiotic treatment group. **c** The boxplot illustrates temporal changes of the alpha diversity in the two diarrhoeal weeks, calculated as the difference in Shannon index between two consecutive timepoints (D7–D1 for week 1; D14–D7 for week 2) and grouped by antibiotic treatment. **d** Intra-patient temporal trend of alpha diversity (Shannon index), grouped by antibiotic treatment. Only patients with samples collected at at least two consecutive time points were included (D1–D7, and D1–D7–D14). Each continuous line represents a patient, coloured by their hospitalisation duration. The dashed line is visualised by fitting a Local Polynomial Regression Fitting (LOESS) on the temporal data. Results from panels **b**–**d** were derived from the longitudinal study only.

The gut microbiota’ alpha diversity, representing species richness and evenness, at diarrhoea onset (D1) was significantly different when comparing between the longitudinal and cross-sectional studies (Wilcoxon signed-rank test, *p*-value = 0.006) (Fig. 1a). While these two groups had the same age-month distribution (Wilcoxon signed-rank test, *p*-value = 0.14), the weight-for-age z-scores were higher in the Longitudinal group (Wilcoxon signed-rank test, *p*-value < 0.001), which might explain the heightened richness observed in patients in this cohort. Once we compared between time points, particularly within the longitudinal study cohort, the microbiota of early diarrhoea onset (D1) showed the lowest diversity (mean of indices: Shannon: 2.41, Chao1: 60.8, Simpson: 0.73; Wilcoxon signed-rank test *p*-values < 0.01) (Fig. 1a). In contrast, diversity was highest on D14 (mean of indices: Shannon: 2.88, Chao1: 76.0, Simpson: 0.83; *p*-values < 0.0001). The control group were age-matched Vietnamese children without diarrhoea in the cross-sectional study, and its alpha diversity was comparative to that of D7 and D14 in the longitudinal study (Tukey posthoc test, *p*-values ranging from 0.24 to 0.69). This implies that diarrhoea caused a transient drop in alpha diversity at onset, which is ameliorated during recovery and approaching that of healthy state at D7 and D14.

To track the temporal change of the microbiota within patients, we only utilised data from the longitudinal study. Patients receiving antibiotics experienced a less pronounced difference in Shannon diversity during the first week (D1–D7) compared to those without treatment (Wilcoxon signed rank test, *p* = 0.004) (Fig. 1c). This suggests that antibiotic usage led to a slower recovery of the gut microbiota diversity initially. However, this was followed by a dramatic increase in diversity for the antibiotic treated patients during the second week (*p* = 0.044). In contrast, the non-antibiotic group’s Shannon index remained relatively static during this period. This observation was again captured when we mapped the changes in alpha diversity within each patient. Antibiotic administration delayed the increase in diversity initially (D7), but diversity eventually increased at D14 to levels similar to those observed in the non-antibiotic group (Fig. 1d). This could be explained by opposite trends of diversity change induced by antibiotics (with nearly equal proportion), resulting in an overall negligible change observed at D7 in treated patients. Furthermore, antibiotic induced a drop in all diversity measures at D7 (but not D14) when compared to the non-antibiotic group, with estimated changes of −9.5 (Chao1), −0.53 (Shannon), and −0.12 (Simpson) (antibiotic treatment:day7, Linear mixed-effects model - LMM, *p*-value < 0.01, Supplementary Table 1).

### The effects of antibiotics on inter- and intra-individual gut microbiota configurations

**Fig. 2:**
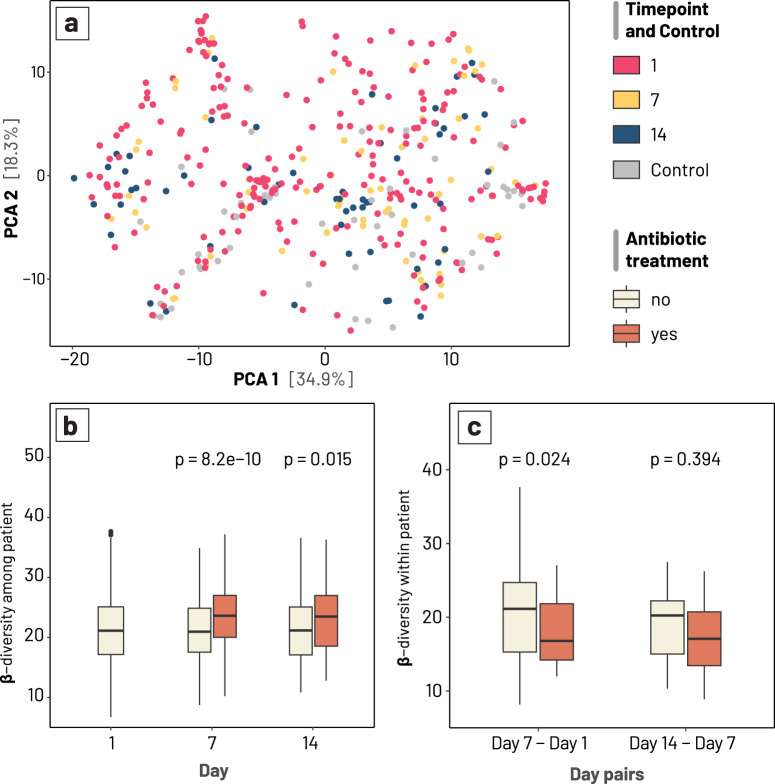
Temporal changes in beta-diversity in the gut microbiome of children recovering from diarrhoea. **a** Principal Coordination Analysis (PCoA), performed on the phylogenetic-assisted isometric log-ratio (PhILR) transformed data (with Euclidean distance). Points are coloured by sampling timepoints (Days 1, 7, and 14 for diarrhoeal gut microbiomes) and non-diarrhoeal controls (grey). **b** Distribution of inter-patient beta-diversity, calculated separately for each sampling timepoints and antibiotic treatment. **c** Temporal changes of intra-patient beta-diversity, calculated as differences between two consecutive timepoints and grouped by antibiotic treatment. *P*-values denote results of statistical significance by Wilcoxon’s signed rank test. Results from panels **b** and **c** were derived from the longitudinal study only.

We next used beta-diversity to explore the dissimilarity in the gut microbiota structure between samples, as well as to map the intra-individual temporal dynamic^24,25^. We transformed the raw count data using the PhILR method, and beta-diversity was calculated using Euclidean distance. We did not observe separate clustering of the microbiota based on sampling time points (Fig. 2a), but these were shown to contribute significantly in explaining the microbiota variation (PERMANOVA, *p* = 0.001 for age-month and sampling day). This likely indicates that microbiota succession varies depending on the patient’s condition, and there was no general configuration denoting the recovering gut microbiota. Likewise, though the beta-diversity calculated between patients (separately for each timepoint) was higher than that of within-patients (conducted for pairs of consecutive time points) (Wilcoxon signed rank test, *p* < 0.001), this difference was negligible. This suggests that intra-individual taxonomical changes during microbiome recovery were nearly as significant as inter-individual variation.

Comparison between time points showed that between-patient differences at D1 were significantly lower than that of D7 and D14 (*p*-values < 0.05), though these differences were small (0.9 and 0.6, respectively). Since treatments (antibiotic, probiotic) and diarrhoea itself could destabilise the gut microbiome in diverging trajectories^14^, the microbiota composition could be more variable in later periods compared to the first day. Indeed, the gut microbiota of patients receiving antibiotics displayed more compositional variation than those without antibiotic use, which was consistent for both D7 and D14 (Wilcoxon signed-rank test, *p*-values < 0.05) (Fig. 2b). Similar to its effect on alpha-diversity, antibiotic treatment resulted in a lesser degree of change in beta-diversity within patients (LMM, antibiotic treatment estimate = −3.63, *p* < 0.01, Supplementary Table 2), particularly visible during the first week (Wilcoxon signed-rank test, *p* = 0.024; Fig. 2c). These findings indicate that antibiotic usage likely leads to a slower recovery in the microbiota composition.

### Antibiotic treatment induces differing patterns of microbiota recovery post-diarrhoea

**Fig. 3:**
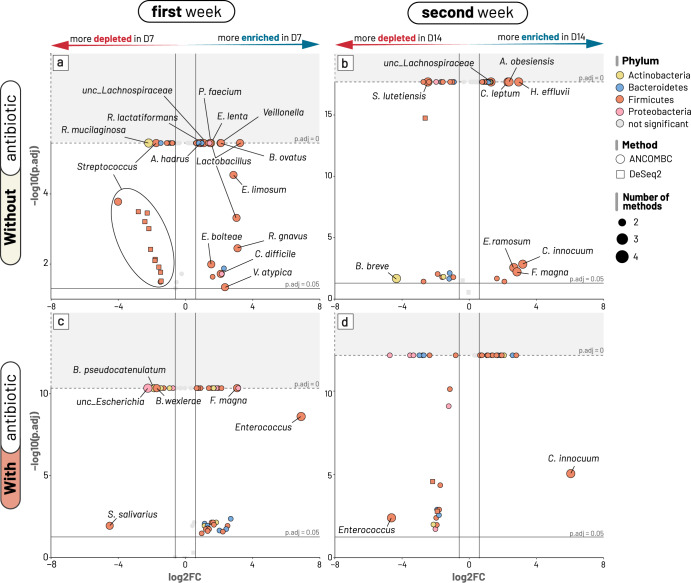
Bacterial taxa showing significant differential abundance during the diarrhoea recovery phase. Panels **a** and **c** illustrate differential abundance between days 7 and 1, while panels **b** and **d** illustrate differential abundance between days 14 and 7 (without and with antibiotic treatment, respectively for each pair). For each panel, OTUs (points) were defined as significant and plotted if they were detected in at least two of the four tested methods (ANCOMBC, DESeq2, MaAsLin2, Limma-Voom; adjusted *p*-values ≤ 0.05). The x-axis denotes the log2 fold change (Log2FC) of OTUs between two examined time points, and the y-axis denotes the negative log10 of the adjusted *p*-value of each comparison. Log2 fold change was preferentially derived from ANCOMBC test output (circle shape), and from DESeq2 (square shape) if ANCOMBC did not generate significant results. The vertical lines delineate log2 fold change of (−0.5) and 0.5, and the horizontal lines delineates adjusted *p*-values at 0.05 and pseudo-value of 0 (broken line; for ANCOMBC test output). OTUs were coloured by phylum, with their size proportional to the number of tests supporting their significant results. For readability, species classification is only labelled to OTUs with significance supported by at least three tested methods. “unc_” stands for unclassified.

**Fig. 4:**
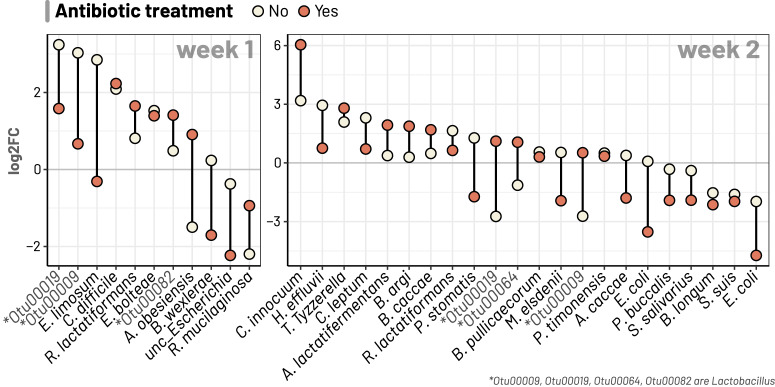
Antibiotic treatment inducing differing effects on abundances of bacterial taxa during diarrhoeal recovery. The two panels respectively show bacterial taxa (OTUs) detected as differentially abundant in both conditions (with and without antibiotic treatment) for comparisons in weeks 1 (D1–D7) and 2 (D7–D14). Log2 fold change (log2FC) was derived from ANCOMBC test output. For each taxon, if the line (connecting log2FC values of two conditions) intersects 0, it is suggested that antibiotic treatment likely induced contrasting effect on the taxon during diarrhoeal recovery.

**Fig. 5:**
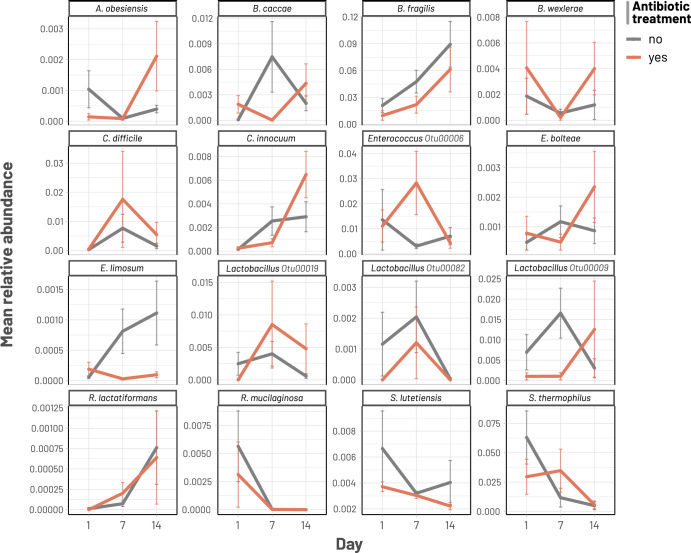
Temporal changes of selected bacterial taxa during diarrhoeal recovery. Multiple line plots illustrate the changes in mean relative abundances of specific taxa, separated by the condition of antibiotic treatment.

We next focus on specific taxonomic changes in gut microbiota during diarrhoea recovery using samples from the longitudinal study. The top four dominant phyla (Firmicutes, Actinobacteria, Bacteroidetes, and Proteobacteria) made up the majority of the gut microbiota (>98% at all three time points) (Fig. 1b). At the genus level, the most abundant genera included *Bifidobacterium*, *Streptococcus*, *Bacteroides*, *Escherichia*, *Veillonella* and *Phocaeicola* (Supplementary Fig. 3). Tracking the temporal trend revealed a marked shift in the phyla’s relative abundance, in which Bacteroidetes (from 8% to 20%) gradually replaced Proteobacteria (from 16% to 6%) to become the third-most prevalent at D14. This was mainly due to the gradual increases in the abundances of *Bacteroides* and *Phocaeicola* post diarrhoea, accompanied by the reducing *Escherichia* (Supplementary Fig. 3). However, these changes were less visible in patients treated with antibiotics, especially during the first week. In order to produce insights into changes at the species/OTU level between time points, we next employed differential abundance (DA) analyses on paired microbiota (from the same patients) to account for inter-individual variation. We separately analysed patients with and without antibiotic treatment to infer the differential effects of such treatment on the microbiota dynamics (Figs. 3–5). Four DA approaches (ANCOMBC, DESeq2, MaAsLin2, and Limma Voom) were employed for each comparison, and only taxa identified by at least two of these approaches were determined as differentially abundant and reported in the following discussion.

Our analyses identified several patterns of gut microbiota succession consistent in both antibiotic and non-antibiotic treatment groups. For instance, taxonomic shifts in the first week were characterised by dramatic reductions of oral or ileal-originated bacteria, including *Streptococcus* spp. (*S. thermophilus*, *S. salivarius, S. lutetiensis*) and *Rothia mucilaginosa* (log2 foldchange of −4.0 to −0.9; Figs. 3a, c and 4)^24^. Such decline was more profound in the non-antibiotic group, exemplified by the higher reduction of *R. mucinlaginosa* (4 folds versus 2 folds) and the exclusive depletion of a major *S. thermophilus* OTU (log2 fold change of −4) found in these patients (Fig. 4, Supplementary Data). Consistent with their changes in relative abundances (Supplementary Fig. 3), Bacteroidaceae commensals (*Bacteroides* and *Phocaeicola*) underwent substantial expansion in the gut microbiota at D7, with increases of *Bacteroides fragilis* (6.5 folds), *Bacteroides uniformis* (5 folds), *Phocaeicola dorei* (2.5 folds), *Phocaeicola vulgatus* (4 folds), and *Bacteroides caccae* (3 folds) (Supplementary Data). Particularly, the proliferation of *B. fragilis* was most visible in terms of relative proportion (Fig. 5). Likewise, commensal anaerobes such as *Clostridioides difficile*, *Enterocloster bolteae*, and *Ruthenibacterium lactatiformans* showed a consistent enrichment in both patient groups at D7 (log2 fold change of 0.8–2.2, Figs. 3a, c and 4, Supplementary Data). Since the majority of our patients (*n* = 85/90) received *Lactobacillus* probiotics for initial treatment, its proliferation was also visible at D7, with more remarkable enrichment in the non-antibiotic group (eight-fold versus three-fold increase; Fig. 4). Nevertheless, the abundance of these taxa decreased at D14 in the non-antibiotic group (log2 fold change of −1.1 to −2.7), while they remained slightly enriched in patients receiving antibiotics up till the second week (log2 fold change of 0.5–1.1) (Fig. 4, Supplementary Data). Recovery in the second week was consistently marked by the continuing decline of dysbiosis-associated *Streptococcus* and *Escherichia* species at D14, (log2 foldchange ranging from −0.04 to −4.7) (Figs. 3b, d and 4). This was accompanied by the expansion of many gut commensals of Lachnospiraceae and Ruminococcaceae taxa, including *Tyzzerella*, *Anaerotignum lactatifermentans*, *Blautia argi*, *Hungatella effluvii*, *Clostridium leptum*, *R. lactatiformans*, and *Butyricicoccus pullicaecorum* (Fig. 4). Notably, *Clostridium innocuum* showed the highest level of enrichment in both patient groups but with much higher foldchange in patients treated with antibiotics (64 versus 8 foldchange**;** Figs. 3b, d and 4). Similarly, though *B. caccae* expanded in both groups, its elevation was more pronounced in the antibiotic group (3 versus 1.4 foldchange, Supplementary Data, Fig. 3).

Alongside the general recovery trends observed in all patients, antibiotic treatment could exert a distinct effect on the gut microbiota, sometimes with opposite impacts on the same taxon. For example, while the short chain fatty acid (SCFA)-producing *Eubacterium limosum* and *Blautia wexlerae* were enriched at D7 for the non-antibiotic group (log2 fold change up to 2.8), their abundance plummeted in the antibiotic treated patients (log2 fold change of −1.7) (Figs. 3a, c, 4 and 5). Moreover, we observed specific effects exclusive to the antibiotic group, such as a four-fold decline in *Bifidobacterium pseudocatenulatum* (*p*-value = 4e−11) at D7 (Fig. 3c), mirroring the general trend of decreasing abundance in *Bifidobacterium* post-D1 (Supplementary Fig. 3). In antibiotic treated patients, the most significant enrichment at D7 was for *Enterococcus* (>32-fold increase, *p*-value = 10^−9^ in all 4 DA methods; Fig. 3c), followed by *Finegoldia magna* (~8-fold increase, Fig. 3c). However, *Enterococcus* abundance was markedly reduced at D14 (log2 foldchange of −4.6; detected by 3 DA methods) (Figs. 3d and 5), demonstrating that its enrichment was probably transient following antibiotic use. Besides, compared to D7, D14 witnessed the diminishing of *Faecalbacterium prausnitzii* and *Prevotella copri* (log2 foldchange of −2.3 and −2.7, respectively) in antibiotic treated patients, coupled with expansions of *Parabacteroides distasonis* and *Collinsella aerofaciens* (log2 foldchange of 2.6 and 2 respectively) (Supplementary Data).

In contrast, for patients without antibiotic use, recovery at D7 was specifically accompanied by substantial increase of SCFA-producing *Bifidobacterium breve*, *Phascolarctobacterium faecium*, and *Ruminococcus gnavus* (Fig. 3a, log2 foldchange from 1.45 to 3), which were not observed in antibiotic treated patients. At D14, the shrinkage of *Bifidobacterium breve* was most noticeable (log2 foldchange of −4.33; Fig. 3b), signifying its transient overabundance in the post-perturbed gut. D14 recovery was also characterised by the substantial rise of *Erysipelatoclostridium ramosum* (6-fold increase) and several Peptoniphilaceae members (*F. magna*, *Anaerococcus obesiensis*, *Peptoniphilus coxii*) (Fig. 3b; Supplementary Data).

## Discussion

Our study provides an understanding of the longitudinal dynamics in gut microbiota as children recover from diarrhoea, and the short-term effects induced by antibiotic treatment. Diarrhoea onset is associated with the elevation of oxygen levels in the gut environment, which enables the proliferation of facultative anaerobes such as *Escherichia* and *Streptococcus*^3,24^. Our findings showed that these taxa, together with oral/ileal-originating bacteria such as *Rothia*, acted as transient colonisers in the gut and are diminished during the first week^26^. Similar to previous findings, recovery post diarrhoea was marked by the expansion of *Bacteroides* and *Phocaeicola* probably due to their expansive repertoires of carbohydrate-degrading enzymes (notably targeting mucins and fibre)^3,11,12^. These catabolic potentials may have allowed Bacteroidetes to capitalise on the host-derived carbon resource in a low-competition environment post-diarrhoea. The microbiota at D7 and D14 were further characterised by the return of anaerobic Firmicutes, including SCFA producers such as *Faecalibacterium prausnitzii*, *Clostridium leptum*, *Eubacterium*, and *Blautia*^22,23^. It was noticeable that probiotic treatment resulted in a transient and low-abundance colonisation of *Lactobacillus*, likely explaining its inefficacy in improving clinical outcomes in our patient cohort.

Significantly, we found that antibiotic treatment delayed the increase in alpha- and beta-diversity within patients during recovery, exemplified by a lesser degree of taxonomic changes at week 1 post-treatment. This was notified as a weaker trend both in the decline of oral-associated taxa (*Rothia*, *Streptococcus*) and the elevation of SCFA-producers (*Blautia*, *Eubacterium*) at D7, possibly signalling a slower rate in gut microbiota recovery in treated patients. Though the rise in diversity eventually approximated that of the non-antibiotic patients in the second week, certain markers of the healthy gut (such as SCFA-producing *F. prausnitzii* and *P. copri*) remained depleted at D14 for treated patients. This suggests that despite the transient effect of antibiotics on gut microbiota diversity, the taxonomic composition may remain divergent to that of non-antibiotic recovery. Interestingly, we found that the common spore-forming *C. difficile* and *E. bolteae* increased in similar foldchange at D7 regardless of antibiotic treatment, and *C. innocuum* experienced the highest foldchange in antibiotic condition at D14. This indicates that the sporobiota are unaffected by antibiotic exposure, and they may even proliferate better in the absence of other competitive commensals, faciliated by antibiotic use^25,27^.

Our findings showed that antibiotic treatment exclusively affected some taxa. The most remarkable was the signature expansion of *Enterococcus* in the early recovery phase following treatment, which is in line with previous research^26,28^. As early as 1978, Goldmann and colleagues have reported notable *Enterococuss* enrichment post-antibiotic exposure in neonates admitted to intensive care^29^. The bloom of this genus, together with other Gram-positive bacteria, was also observed following treatment with amoxicillin-clavulanate in the previous research^30^. Furthermore, treatment with most classes of bactericidal antibiotics, including extended-spectrum cephalosporins and fluoroquinolones as administered in our cohort, has been associated with *Enterococcus* overabundance^31,32^. This could be explained by the intrinsic or sporadic resistance in *Enterococcus* to most commonly used antibiotics, aiding its survival and replication in the gut environment created by antibiotic clearance^19,33,34^. Similar to previous reports, our findings illustrated that *Enterococcus* dominance appears transient and diminished markedly during the second week^31,35^. Though this transient colonisation might not affect the patient’s clinical outcomes, its overgrowth could instigate horizontal transfer of AMR genes to other gut commensals^18^. Multiple AMR determinants in *Enterococcus* are known to be mobilised by plasmids or transposons^19^. Additionally, *Enterococcus* has immunomodulatory properties that support the metabolism of nutrients in the gut, but also possesses complex virulence traits^36,37^. Thus, the overabundance of *Enterococcus* post-antibiotic warrants more future in-depth research to understand its impact on gut health. On the other hand, it was noted that *B. pseudocatenulatum* was the most susceptible member of *Bifidobacterium* to antibiotics. Our previous research has shown that this species was prevalent and abundant in the gut microbiotas of Vietnamese children^38^, and it contributes to gut health through the degradation of complex plant-based carbohydrates and ameliorates pro-inflammation responses^39,40^. Thus, its shrinkage could lead to deleterious consequences on gut health.

Moreover, antibiotics could act as a selective factor in differentiating the gut microbiota configuration at the species or strain level. Particularly, *Bacteroides* abundance was shown to increase after a course of first/second generation of cephalosporins or amoxicillin/clavulanate^14,41^. Still, its abundance would decrease initially following treatment with carbapenems or cephalosporins of later generations^14,42^. Besides, Our findings highlight that several *Bacteroides* OTUs, classified as *B. fragilis*, *B. uniformis*, and *B. faecichinchillae*, showed remarkable increases at D7 post-antibiotic treatment, while *B. cacae* and *P. vulgatus* were more enriched in the non-antibiotic group. Differing mucin-degrading capability and AMR profiles among the *Bacteroides/Phocaeicola* genomes probably determine which species or strains could persist and thrive during the early recovery phase^43–45^. Variable distribution, even at the subspecies level, in key enzymes acting on terminal mucin capping structures, including sulfatases and specific glycosyl hydrolases (GH33: sialidase; GH29 & GH95: fucosidases) have been observed in *Bacteroides*/*Phocaeicola* genera^46–48^. Besides, AMR determinants in *Bacteroides* were recently shown to mobilise via integrative conjugative elements^49^. Therefore, it is difficult to precisely predict which taxa could predominate in the gut microbiota since the accessory genome could significantly contribute to their competitive advantages. Metabolic activeness of the *Bacteroides*/*Phocaeicola* genera could then contribute positively to the growth of other commensals. Particularly, sialic acid released from the host mucus by *B. thetaiotaomicron* served as a substrate for developing *C. difficile* in mouse models^50^, which could explain *C. difficile* overabundance at D7 in our patient cohort.

In summary, we evaluated the changes in diarrhoeal gut microbiota that occur following the treatment of watery diarrhoea with antibiotics. Given the probiotic trial design and the consistent treatment guideline implemented in a single hospital, we envision that the effects generated by other factors (rehydration, nutrition, different infection aetiologies, etc.) on the gut microbiota were less significant compared to antibiotic treatment. This study is limited to taxonomic profiling in only three consecutive time points. Future approaches should utilise well-designed longitudinal clinical cohorts, with denser sampling time points, longer follow-up timeframe, and high-resolution shotgun metagenomic or long read sequencing, to fully characterise the correlation between gut microbiome dynamic and clinical outcomes.

## Methods

### Sample collection and DNA sequencing

The samples used in this study were taken from a previously published double-blind, randomised, placebo-controlled trial of *Lactobacillus acidophilus* for treatment of acute watery diarrhoea (trial number: ISRCTN/ISRCTN88101063, approved by Ho Chi Minh City Children Hospital no. 2 and the Oxford Tropical Research Ethics Committee)^21^. Vietnamese children aged 9 to 60 months were hospitalised with acute watery diarrhoea with a history of less than three days and were recruited into the trial upon obtaining written informed consent from their parents/guardians. In this study, acute watery diarrhoea was defined as the passage of loose or watery stools at least three times in 24 h but did not contained blood or mucus in the past three days. The participants were followed up for two weeks, and stool samples were collected at three-time points: day 1 (at admission), day 7, and day 14. The trial enroled 300 patients. For this study, we selected data and samples from 85 patients from the probiotic arm to minimise treatment variability and to investigate the colonisation efficacy of the probiotic. We also included five patients from the non-probiotic arm, of which three received antibiotics, to increase the number of antibiotic-treated patients but also control for treatment variability. We preferentially selected patients who had samples at either two or three-time points, resulting in a total of 218 stool samples for microbiome sequencing.

DNA extraction was performed using the FastDNA SPIN Kit for Soil (MP Biomedicals, California, USA), following the manufacturer’s protocol. Extracted DNA was transferred to the Genome Institute of Singapore (GIS) for 16S rRNA sequencing. All DNA samples were amplified using PCR and the 338F-1061R primer set in order to achieve high taxonomic resolution up to the species level. This process resulted in >700 bp amplicons, which cover a region from V3 to V6 of 16S rRNA and could be assembled to retrieve >92% of the sequences in the Greengenes database^51^. PCR products were cleaned with 1X AMPURE beads and randomly fragmented using Covaris (model LE220) to obtain fragments of ~200 bp. GeneRead DNA Library I Core Kit (Qiagen, Germany) was used to prepare a library subjected to sequencing on an Illumina HiSeq2500 platform, producing 75 bp paired-end reads. Additionally, we included published sequence data from a cross-sectional study investigating the gut microbiota perturbation in Vietnamese children with diarrhoea, which was also generated by the aforementioned methodology. This dataset consists of 200 microbiome samples (55 controls, 145 diarrhoeal cases) collected from children under five years-old^12^.

### Assembly of 16 rRNA sequences and OTU clustering

We pooled sequencing data from the probiotic trial and cross-sectional studies for analysis. A pre-computed database containing SILVA 16S rRNA SSU (small subunit) sequences clustered at 97% similarity was used as the ref. 52 For each sample library, Sickle was used to remove read pairs with quality <30 and length <60^53^. The resulting library and the modified SILVA database were used as inputs for EMIRGE (Expectation Maximisation Iterative Reconstruction of Genes from the Environment) to reconstruct full-length 16S rRNA SSU DNA sequences with 40 iterations and a joining threshold of 97%^54,55^. For each library, this process produced a set of assembled and clustered lines, each with its corresponding abundance. Sequences with sample-wise normalised relative abundances less than 0.001% were removed from further analysis. By scaling the number of successfully mapped reads to the relative abundance of each sequence assembled by EMIRGE, pseudo counts of each sequence were obtained. Assembled sequences were aligned to the SILVA database, generating an alignment of sequences containing only the amplified region (388F-1061R). Only gap columns were eliminated, and sequences with more than eight ambiguous sites were removed. For the remaining sequences, ambiguous sites were assigned random nucleotides (A, T, G, C). Full sequence DECIPHER was utilised to detect and remove potential chimeric sequences based on the ‘gold’ database^56^. Furthermore, the resulting sequences were subject to self-BLAST to identify and remove sequences with terminal repeats (low-quality assemblies). The remaining non-chimeric sequences from all samples, as well as their corresponding abundances, were input into vsearch for clustering at 97% similarity^57^. OTUs (Operational Taxa Units) with the sum of sample-wise relative abundances less than 1% were further removed, resulting in 1,904 most prevalent OTUs across 416 samples. Ribosomal Database Project (RDP) classifier v1.36.0 was used to assign the taxonomy of OTUs representatives up to genus level, with a minimum support threshold of 80%^58^.

### Microbiota data analytics

The OTU table, taxonomic classification and associated metadata were combined into a phyloseq object^59^. Samples without associated metadata (*n* = 6) were removed, resulting in 410 samples with 1,886 taxa (Longitudinal study: 218 samples; Cross-sectional study: 192 samples). All downstream analyses were conducted in R Studio (version 4.2.1) with the base functions and multiple packages such as phyloseq, dplyr, vegan, ANCOMBC, DeSeq2, ggplot2, and other packages^59–62^. During preliminary analysis, we detected the presence of many bacteria belonging to the oral microbiome in our samples. These OTUs shared high nucleotide similarity (by BLAST) with the references deposited in the expanded Human Oral Microbiome Database (eHOMD; www.homd.org). Previous studies also showed that the microbiome of the small intestine shares more similarities with the oral cavity, such as the overabundance of *Streptococcus*, *Rothia*, and *Actinomyces*^24^. Therefore, we divided the OTU collection based on their likely niches: (1) colonic (*n* = 1734) and (2) oral and small intestinal (*n* = 152), and the two alignments were conducted using PASTA^63^. Subsequently, phylogenetic reconstruction was performed independently for each alignment using IQ-TREE, with default parameters and 1000 ultrafast bootstraps^64^. These two trees were then joined at the basal internal node to form the resulting phylogeny used for downstream analyses and were used in the PhILR transformation of raw count data.

Alpha diversity illustrates the richness of the microbiota inside each sample, which would help us track the changes in microbial diversity during a diarrhoea episode^65^. In this study, three indices (Shannon, Simpson, and Chao1) were used to calculate the alpha diversity of the microbiota, using the function ‘estimate_richness’ implemented in the package phyloseq. The measurements were then tested with analysis of variance (ANOVA) and Tukey post-hoc test (function ‘TukeyHSD’ in ‘stats’ R package) to analyse the statistical differences among time points and between the two antibiotic usage groups.

### Principal component analysis (PCA)

Since microbiota data generally have a high degree of sparsity, we conducted preliminary filtering and normalisation prior to visualisation by PCA. Firstly, singleton OTUs were removed, resulting in 1,292 OTUs retained in the filtered phyloseq object. We next performed imputation, using “mbImpute”, to reduce the non-biological zeros and improve the performance of differential abundance analyses (ANCOMBC and DESeq2)^66^. The “mbImpute” used the OTU table, the sample data, and the pairwise phylogenetic distance to decrease the sparsity of the OTU table. Wrench was then used to normalise the OTU count table. This method generalises zero-inflated data for differential abundance analysis^67^, and it has been reported to outperform other normalisation methods at all sparsity levels to handle systematic biases^68^. The normalised OTU count table was transformed by phILR (“philr” package) using default parameters, which utilised their phylogenetic relationship^62^. This transformation created a table of isometric log-ratio (ILR) “balances”, calculated as the log-ratios of the geometric means of the relative abundances between two clades of taxa^62^. The resulting “balances” were used to calculate an Euclidean distance matrix, which was used for ordination using principal component analysis (PCA). This method has been reported to be more suitable for the compositional nature of microbiome data, and outperform other transformation approaches^62,69^.

### Statistical analyses

Multivariate analysis was used to identify the predictors significantly associated with differences in clinical outcomes and microbiota configurations. For clinical data, the response variable (hospitalisation days) was fit into a multiple linear regression model with the predictors for each patient: “hospitalization_days ~ sex + wfa_zscore + age_month + Infection_type + breastfeeding + antibiotic_trt”. For microbiota data, permutational multivariate analysis of variance (PERMANOVA; implemented in the function ‘adonis2’) was used to test the association of the aforementioned demographic and clinical covariates, together with sampling day, with the microbiota composition (using different distance matrices): “distance_matrix ~ age_month + sex + wfa_zscore + day + breastfeeding”. PERMANOVA is a non-parametric test that better suits the non-normally distributed microbiota data. To account for intra-patient changes in alpha (Shannon, Chao1, Simpson indices) and beta (PhILR-transformed Euclidean distances) diversities, these diversity measurements were independently treated as the response variable and fit into a linear mixed effects model (function ‘lmer’ in package ‘lme4’), with the aforementioned clinical/demographic and the interaction term ‘day*antibiotic’ serving as predictors, and patient ID as a random factor: “diversity index or beta-diversity distance ~ sex + age_month + wfa_zscore + Infection_type + day*antibiotic_trt + (1|patient_ID)”.

### Differential abundances analysis

The absence of microbiome clustering based on sampling days motivated us to conduct more in-depth differential abundance analyses. We focused on 218 samples of the longitudinal study, and split the dataset based on the usage of antibiotics and consecutive time points, generating four comparison groups (D1–7 with antibiotics, D1–7 without antibiotics, D7–14 with antibiotics, and D7–14 without antibiotics). D1–7 subset includes 134 samples of 67 patients, in which patients have reported two time points (D1 and D7). Similarly, the D7–14 subset had 76 samples (38 patients). We performed differential abundance analyses to determine the significant changes in OTU abundances in each comparison group. Rare taxa, with prevalence less than 5% of patients per comparison group, were removed for downstream analyses. The filtered dataset still retained more than 90% of OTUs of the unfiltered data. Since differential abundance analysis is prone to generate false positive findings, we conducted these analyses independently using four R packages (ANCOMBC, DESeq2, MaAsLin2, and Limma Voom), with correction for multiple hypothesis testing applied to each method. For all comparisons, the model design was set to “~ Patient + day”, with the reduced model as “~Patient” (DESeq2) or grouped by “day” (ANCOMBC), to account for variations observed within individual patients, and taxa with adjusted *p*-value ≤ 0.05 (and base mean >30 for DESeq2) were considered significant hits. For ANCOMBC, we input the unnormalised OTU count data with set parameters: prevalence cut at 0.001, library cut at 1000, and keeping structural zeros. For DESeq2, since our data is highly sparse, we prepared the input by applying the zero-inflated NB model implemented in the zinbwave packages^70^. The analysis was conducted with a likelihood ratio test (LRT), “local” fit type, and minReplicatesForReplace = 7. For MaAsLin2, the input was a Wrench-normalised OTU count table with default settings but without filtering for OTU abundance, prevalence, and application of other normalisations. For Limma-Voom, we used the same input OTU count table as for DESeq2, except for scaling down library sizes for TMMwsp normalisation. For each comparison, only significant OTUs identified by at least two of the aforementioned approaches were determined as differentially abundant and retained for downstream interpretation and visualisation. Our results showed that all significant OTUs (per comparison group) were covered either by ANCOMBC or DESeq2, so estimated foldchanges and relevant statistics from these two methods were preferentially used for summary and visualisation.

### Reporting summary

Further information on research design is available in the Nature Research Reporting Summary linked to this article.

## Supplementary information

Supplementary information

Supplementary Data

Reporting Summary

## Data Availability

Raw sequence data are available in the National Center for Biotechnology Information (NCBI) database under the project number PRJNA1055326.

## Code availability

Source data and R codes used for analysis and visualisation are deposited in Github (https://github.com/lhsnam/longitudinal_microbiome_2023).
