# Supplementary material for: The impact of antibiotics on the gut microbiota of children recovering from watery diarrhoea
Source: NPJ Antimicrob Resist. 2024 Apr 22;2:12. doi: 10.1038/s44259-024-00030-x (PMC11057199; doi:10.1038/s44259-024-00030-x)
Supplement: Supplementary file 1 — Supplementary information [file 44259_2024_30_MOESM1_ESM.pdf]

Supplementary Information

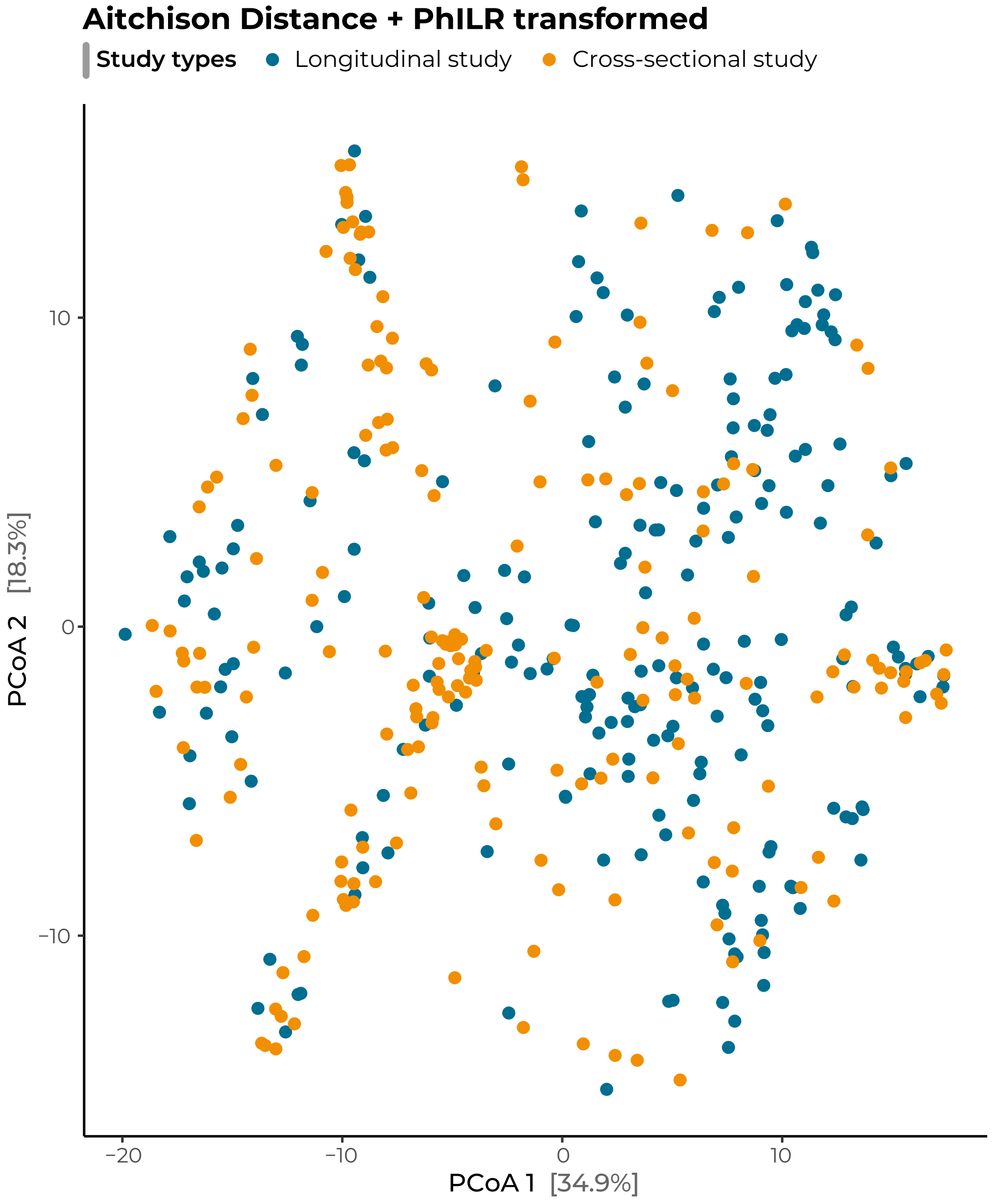

Supplementary Figure 1 Microbiome data were not clustered based on different studies. Quality check upon combining the dataset from the longitudinal and cross-sectional diarrheal microbiome studies. Principal Coordination Analysis (PCoA) conducted on phylogenetic-assisted isometric log-ratio (PhILR) transformed data (with Euclidean distance), with samples coloured by studies.

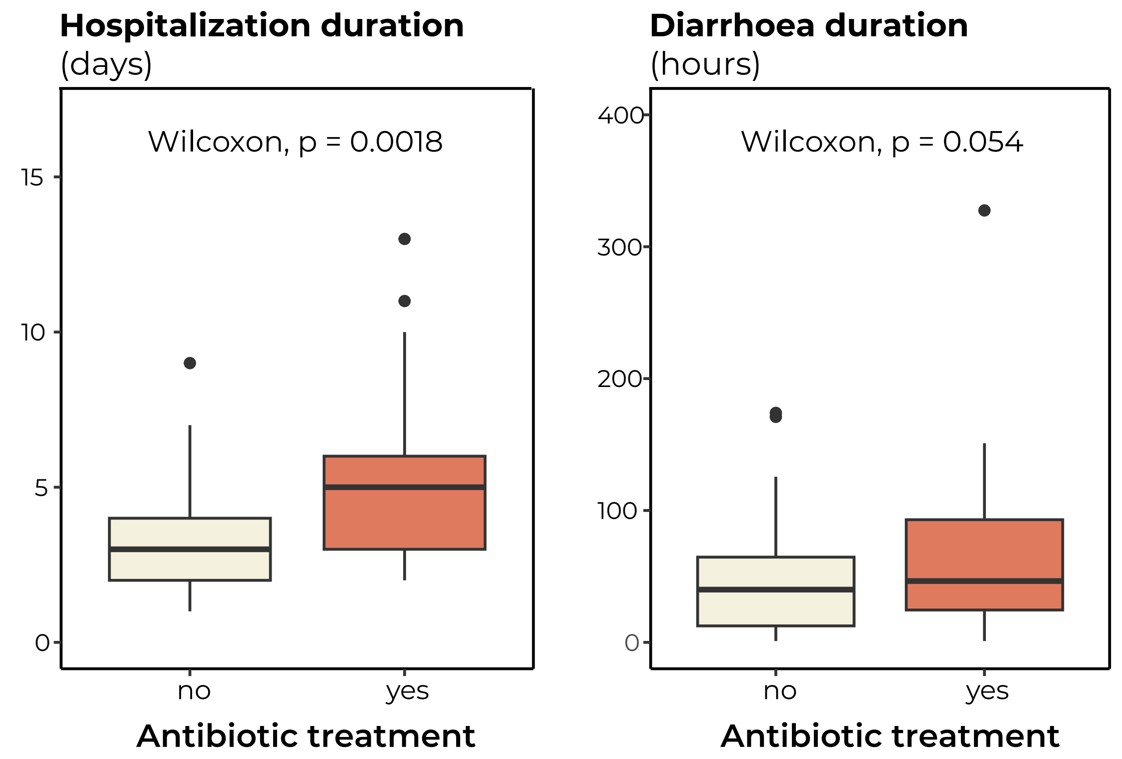

Supplementary Figure 2 Effect of antibiotic treatment on clinical outcomes. The boxplots illustrate the distribution of hospitalization duration (days) and diarrhoea duration (hours) classified by antibiotic treatment.

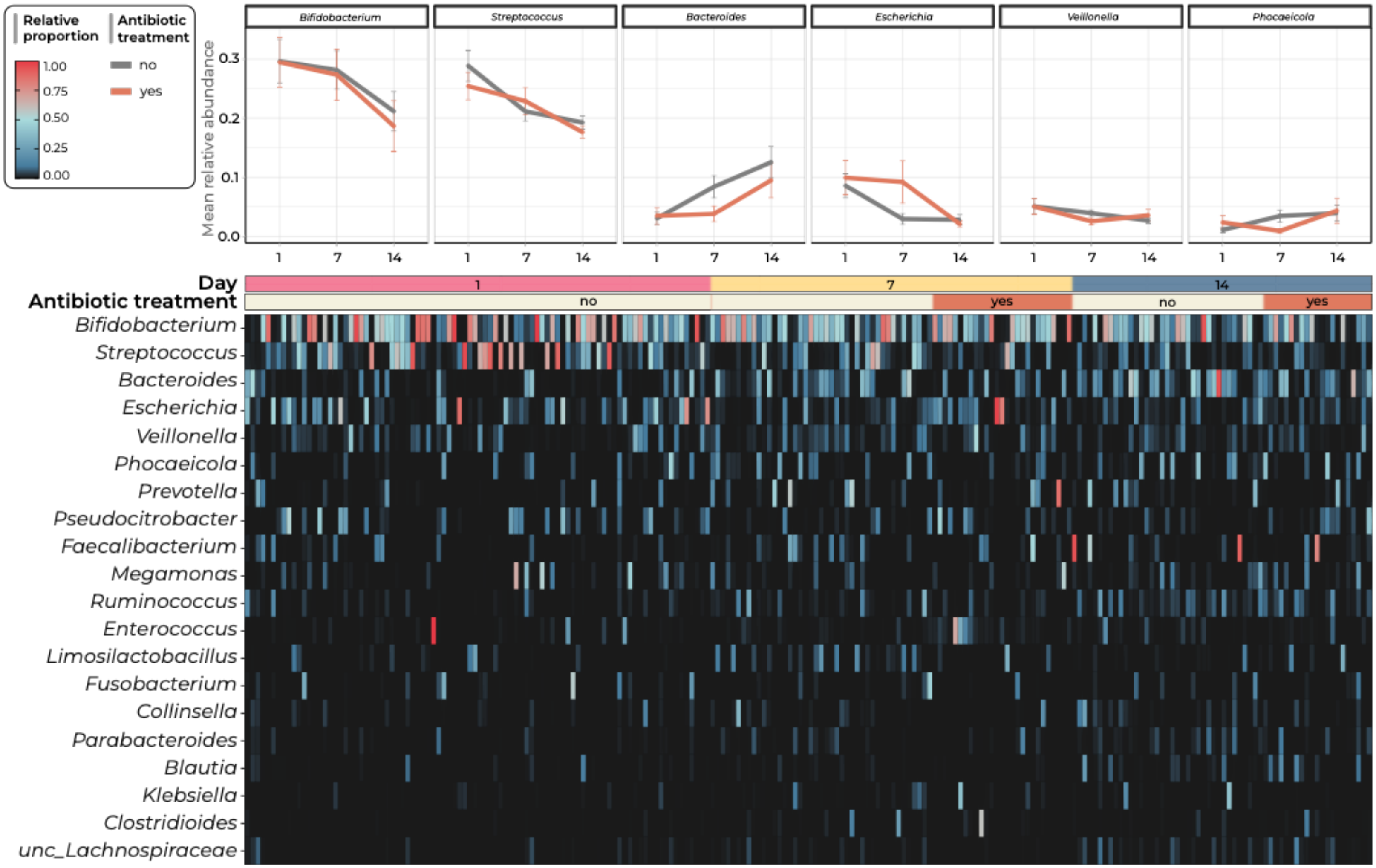

Supplementary Figure 3 Taxonomic composition (at genus level) of the gut microbiome in children recovering from diarrhoea, with data taken from the longitudinal study. The line plots depict temporal changes in relative abundances of the top 6 most abundant genera, classified by usage of antibiotic during treatment. The heat map represents taxonomic composition for each microbiome sample (per column; at genus level), grouped by sampling day and antibiotic treatment. Each cell's colour denotes the relative proportion of a specific genus in the sample (see Legend).

Supplementary Table 1: Change in α-diversity over time in longitudinal subset after adjusting for gender, age month, wfa z-score, infection types, day, and antibiotic treatment<br>lmer(Chao1 ~ sex + age_month + wfa_zscore + Infection_type + day*antibiotic_trt + (1|patient_ID))<br>lmer(Shannon~ sex + wfa_zscore + age_month + Infection_type + day*antibiotic_trt + (1|patient_ID))<br>lmer(Simpson ~ sex + wfa_zscore + age_month + Infection_type + day*antibiotic_trt + (1|patient_ID))				
**Term**	**Estimate**	**Standard error**	**Statistic**	**p-value**
**Chao1 index**				
(Intercept)	51.550	4.544	11.344	0.0000
**Sex [M]**	-3.322	1.823	-1.822	0.0721
**age_month**	0.515	0.082	6.243	0.0000
**wfa_zscore**	-0.311	0.557	-0.558	0.5783
**Infection_type [mixed]**	4.098	3.923	1.045	0.2993
**Infection_type [unknown]**	0.791	4.583	0.173	0.8634
**Infection_type [virus_only]**	3.554	3.848	0.924	0.3584
**Day [7]**	6.748	2.148	3.142	0.0021
**Day [14]**	13.440	2.256	5.957	0.0000
**antibiotic_trt [yes]**	1.079	2.468	0.437	0.6625
**antibiotic_trt [yes] : Day [7]**	-9.497	3.451	-2.752	0.0067
**antibiotic_trt [yes] : Day [14]**	-2.476	3.702	-0.669	0.5046
**Shannon index**				
(Intercept)	2.211	0.191	11.595	0.0000
**Sex [M]**	-0.056	0.076	-0.739	0.4620
**age_month**	-0.006	0.023	-0.246	0.8066
**wfa_zscore**	0.014	0.003	4.144	0.0001
**Infection_type [mixed]**	-0.065	0.164	-0.400	0.6906
**Infection_type [unknown]**	0.014	0.191	0.073	0.9417
**Infection_type [virus_only]**	0.023	0.161	0.140	0.8889
**Day [7]**	0.397	0.097	4.089	0.0001
**Day [14]**	0.344	0.102	3.376	0.0010
**antibiotic_trt [yes]**	0.104	0.108	0.965	0.3356
**antibiotic_trt [yes] : Day [7]**	-0.532	0.156	-3.410	0.0009
**antibiotic_trt [yes] : Day [14]**	0.018	0.167	0.106	0.9156

Supplementary Table 1 (cont.): Change in α-diversity over time in longitudinal subset after adjusting for gender, age month, wfa z-score, infection types, day, and antibiotic treatment				
**Term**	**Estimate**	**Standard error**	**Statistic**	**p-value**
**Simpson index**				
(Intercept)	0.736	0.043	17.027	0.0000
**Sex [M]**	-0.008	0.017	-0.490	0.6244
**age_month**	-0.003	0.005	-0.611	0.5421
**wfa_zscore**	0.002	0.001	2.848	0.0048
**Infection_type [mixed]**	-0.037	0.037	-1.001	0.3179
**Infection_type [unknown]**	0.005	0.043	0.110	0.9127
**Infection_type [virus_only]**	-0.008	0.036	-0.219	0.8266
**Day [7]**	0.101	0.024	4.269	0.0000
**Day [14]**	0.067	0.025	2.701	0.0075
**antibiotic_trt [yes]**	0.037	0.025	1.468	0.1437
**antibiotic_trt [yes] : Day [7]**	-0.117	0.038	-3.082	0.0023
**antibiotic_trt [yes] : Day [14]**	-0.007	0.041	-0.169	0.8659

Supplementary Table 2: Differences of β-diversity over time in longitudinal subset adjusting for gender age month, wfa z-score, infection types, day, and antibiotic treatment<br>lmer(beta_diversity_distance ~ sex + wfa_zscore + age_month + Infection_type + distance_day*antibiotic_trt + (1|patient_ID))				
**Term**	**Estimate**	**Standard error**	**Statistic**	**p-value**
(Intercept)	22.563	3.295	6.848	0.0000
**Sex [M]**	-1.637	1.206	-1.357	0.1803
**age_month**	-0.187	0.400	-0.469	0.6414
**wfa_zscore**	0.103	0.059	1.729	0.0904
**Infection_type [mixed]**	-3.967	2.950	-1.345	0.1851
**Infection_type [unknown]**	-2.953	3.405	-0.867	0.3904
**Infection_type [virus_only]**	-1.574	2.851	-0.552	0.5835
**Period [distance 7-14]**	-2.284	1.100	-2.075	0.0444
**antibiotic_trt [yes]**	-3.633	1.330	-2.732	0.0077
**Period [distance 7-14] : antibiotic_trt [yes]**	1.117	1.857	0.602	0.5506
