# Supplementary material for: The impact of antibiotics on the gut microbiota of children recovering from watery diarrhoea
Source: NPJ Antimicrob Resist. 2024 Apr 22;2:12. doi: 10.1038/s44259-024-00030-x (PMC11057199; doi:10.1038/s44259-024-00030-x)
Supplement: Supplementary file 3 — Reporting Summary [file 44259_2024_30_MOESM3_ESM.pdf]

nature portfolio

Corresponding author(s): Hao Chung The

Last updated by author(s): 2nd February 2024

Reporting Summary

Nature Portfolio wishes to improve the reproducibility of the work that we publish. This form provides structure for consistency and transparency in reporting. For further information on Nature Portfolio policies, see our Editorial Policies and the Editorial Policy Checklist.

Statistics

For all statistical analyses, confirm that the following items are present in the figure legend, table legend, main text, or Methods section.

n/a	Confirmed	
☐	☒	The exact sample size (*n*) for each experimental group/condition, given as a discrete number and unit of measurement
☐	☒	A statement on whether measurements were taken from distinct samples or whether the same sample was measured repeatedly
☐	☒	The statistical test(s) used AND whether they are one- or two-sided<br>*Only common tests should be described solely by name; describe more complex techniques in the Methods section.*
☐	☒	A description of all covariates tested
☐	☒	A description of any assumptions or corrections, such as tests of normality and adjustment for multiple comparisons
☐	☒	A full description of the statistical parameters including central tendency (e.g. means) or other basic estimates (e.g. regression coefficient) AND variation (e.g. standard deviation) or associated estimates of uncertainty (e.g. confidence intervals)
☒	☐	For null hypothesis testing, the test statistic (e.g. *F*, *t*, *r*) with confidence intervals, effect sizes, degrees of freedom and *P* value noted<br>*Give P values as exact values whenever suitable.*
☒	☐	For Bayesian analysis, information on the choice of priors and Markov chain Monte Carlo settings
☒	☐	For hierarchical and complex designs, identification of the appropriate level for tests and full reporting of outcomes
☒	☐	Estimates of effect sizes (e.g. Cohen's *d*, Pearson's *r*), indicating how they were calculated

Our web collection on statistics for biologists contains articles on many of the points above.

Software and code

Policy information about availability of computer code

Data collection	Illumina HiSeq 2500 basecalling software
Data analysis	EMIRGE, SILVA, DECIPHER, BLAST, RDP classifier v1.36.0, phyloseq, dplyr, vegan, ANCOMBC, DESeq2, ggplot2, PASTA, IQ-tree, PhILR, adonis2, lme4, MaAsLin2, Limma Voom

For manuscripts utilizing custom algorithms or software that are central to the research but not yet described in published literature, software must be made available to editors and reviewers. We strongly encourage code deposition in a community repository (e.g. GitHub). See the Nature Portfolio guidelines for submitting code & software for further information.

Data

Policy information about availability of data

All manuscripts must include a data availability statement. This statement should provide the following information, where applicable:

- Accession codes, unique identifiers, or web links for publicly available datasets
- A description of any restrictions on data availability
- For clinical datasets or third party data, please ensure that the statement adheres to our policy

Raw sequence data are available in the National Center for Biotechnology Information (NCBI) database under the project number PRJNA1055326.

Research involving human participants, their data, or biological material

Policy information about studies with human participants or human data. See also policy information about sex, gender (identity/presentation), and sexual orientation and race, ethnicity and racism.

Reporting on sex and gender	Sex and gender information was collected, but these were not relevant to the study's objective.
Reporting on race, ethnicity, or other socially relevant groupings	Information on race/ethnicity or social status were not relevant to the study's objective, and these were not collected.
Population characteristics	The study utilized amplicon metagenomic sequencing on stool samples collected from 90 Vietnamese children (age <5) recovering from watery diarrhoea.
Recruitment	Participants were recruited by study doctors at Children Hospital No. 2, Ho Chi Minh City, Vietnam. There is no identified self-bias in the selection
Ethics oversight	Children Hospital no. 2 (HCMC, Vietnam), and the University of Oxford Tropical Research Ethics Committee (18/04/2012, ref. 14-12). The trial is registered in ISRCTN.com under ISRCTN88101063.

Note that full information on the approval of the study protocol must also be provided in the manuscript.

Field-specific reporting

Please select the one below that is the best fit for your research. If you are not sure, read the appropriate sections before making your selection.

☒ Life sciences ☐ Behavioural & social sciences ☐ Ecological, evolutionary & environmental sciences

For a reference copy of the document with all sections, see nature.com/documents/nr-reporting-summary-flat.pdf

Life sciences study design

All studies must disclose on these points even when the disclosure is negative.

Sample size	85 patients were selected from the probiotic arm (n=150) to minimize treatment variability and to investigate the colonization efficacy of the probiotic. Additional five patients from the non-probiotic arm, of which three received antibiotics, to increase the number of antibiotic-treated patients.
Data exclusions	We excluded patients (the probiotic arm) with stool sample collected from only one time point.
Replication	Data analysis was included in details in the Methods section. Raw data were uploaded to public repository.
Randomization	Randomization was not relevant in this study
Blinding	Blinding was not relevant in this study

Reporting for specific materials, systems and methods

We require information from authors about some types of materials, experimental systems and methods used in many studies. Here, indicate whether each material, system or method listed is relevant to your study. If you are not sure if a list item applies to your research, read the appropriate section before selecting a response.

Materials & experimental systems

n/a	Involved in the study	
☒	☐	Antibodies
☒	☐	Eukaryotic cell lines
☒	☐	Palaeontology and archaeology
☒	☐	Animals and other organisms
☐	☒	Clinical data
☒	☐	Dual use research of concern
☒	☐	Plants

Methods

n/a	Involved in the study	
☒	☐	ChIP-seq
☒	☐	Flow cytometry
☒	☐	MRI-based neuroimaging

Clinical data

Policy information about clinical studies

All manuscripts should comply with the ICMJE guidelines for publication of clinical research and a completed CONSORT checklist must be included with all submissions.

Clinical trial registration	ISRCTN88101063
Study protocol	10.1186/1745-6215-14-27
Data collection	Data were collected from young children (9 - 60 months old) with watery diarrhoea, who were recruited at Children Hospital No. 2 (HCMC, Vietnam) from September 2014 to September 2015
Outcomes	Primary outcome: The time from the first dose of study medication to the start of the first 24 hour diarrhoea free-period as assessed by the parents/guardians

Plants

Seed stocks	*Report on the source of all seed stocks or other plant material used. If applicable, state the seed stock centre and catalogue number. If plant specimens were collected from the field, describe the collection location, date and sampling procedures.*
Novel plant genotypes	*Describe the methods by which all novel plant genotypes were produced. This includes those generated by transgenic approaches, gene editing, chemical/radiation-based mutagenesis and hybridization. For transgenic lines, describe the transformation method, the number of independent lines analyzed and the generation upon which experiments were performed. For gene-edited lines, describe the editor used, the endogenous sequence targeted for editing, the targeting guide RNA sequence (if applicable) and how the editor was applied.*
Authentication	*Describe any authentication procedures for each seed stock used or novel genotype generated. Describe any experiments used to assess the effect of a mutation and, where applicable, how potential secondary effects (e.g. second site T-DNA insertions, mosiacism, off-target gene editing) were examined.*
